# Competitive pricing on online markets: a literature review

**DOI:** 10.1057/s41272-022-00390-x

**Published:** 2022-06-14

**Authors:** Torsten J. Gerpott, Jan Berends

**Affiliations:** University of Duisburg-Essen, Duisburg, Germany

**Keywords:** Competition, Competitive pricing, Dynamic pricing, e-commerce, e-tail, Online markets

## Abstract

Past reviews of studies concerning competitive pricing strategies lack a unifying approach to interdisciplinarily structure research across economics, marketing management, and operations. This academic void is especially unfortunate for online markets as they show much higher competitive dynamics compared to their offline counterparts. We review 132 articles on competitive posted goods pricing on either e-tail markets or markets in general. Our main contributions are (1) to develop an interdisciplinary framework structuring scholarly work on competitive pricing models and (2) to analyze in how far research on offline markets applies to online retail markets.

## Introduction

Setting prices relative to competitors, i.e., competitive pricing,^1^ is a classical marketing problem which has been studied extensively before the emergence of e-commerce (Talluri and van Ryzin 2004; Vives 2001). Although literature on online pricing has been reviewed in the past (Ratchford 2009), interrelations between pricing and competition were rarely considered systematically (Li et al. 2017). As less than 2% of high-impact journal articles address pricing issues (Toni et al. 2017), pricing strategies do not receive proper research attention according to their practical relevance. This research gap holds even more for competitive pricing. In the past, the monopolistic assumption that demand for homogeneous goods mostly depends on prices set by a single firm may have been a viable simplification since price comparisons were difficult. Today, consumer search costs^2^ shrink as the prices of most goods can be compared on relatively transparent online markets. Therefore, demand is increasingly influenced by prices of competitors which therefore should not be ignored (Lin and Sibdari 2009).

In the early 1990s, few people anticipated that business-to-consumer (B2C) online goods retail markets^3^ would develop from a dubious alternative to conventional “brick-and-mortar” retail stores to an omnipresent distribution channel for all kinds of products in less than two decades (Balasubramanian 1998; Boardman and McCormick 2018). In 2000, e-commerce accounted for a mere 1% of overall retail sales. In 2025, e-retail sales are projected to account for nearly 25% of global retail sales (Lebow 2019). Traditional offline channels are nowadays typically complemented by online technologies (Gao and Su 2018). With digitization of various societal sectors in general and the COVID-19 pandemic in particular, the shift toward online channels is unlikely to stop in the future. Besides direct online shops, two-thirds of e-commerce sales are sold through online marketplaces/platforms like Alibaba, Amazon or eBay (Young 2022). The marketplace operator acts as an intermediary (two-sided platform) who matches demand (online consumers) with supply (retailers). Whereas the retailer retains control over product assortment and prices, he has to pay a commission to the marketplace operator (Hagiu 2007). However, these intermediaries often act as sellers themselves, thereby posing direct competition to retailers who have to decide between direct or marketplace channels (Ryan et al. 2012).

Online consumer markets fundamentally differ from offline settings (Chintagunta et al. 2012; Lee and Tan 2003; Scarpi et al. 2014; Smith and Brynjolfsson 2001). Factors which make competition even more prevalent for online than for offline markets are summarized in Table 1.

**Table 1:** Differences of online and offline markets with relevance for the intensity of market competition

	Consumer-related peculiarities (demand side)	Firm-related peculiarities (supply side)
Online	No possibility to physically inspect products before buying Easy comparison of products from various sellers Lowered search costs Increased price transparency Intensified competition (Balakrishnan et al. 2014; Cao and Gruca 2003; Dzyabura et al. 2019; Fisher et al. 2018; Frambach et al. 2007; Yang and Xia 2013)	Possibility to extensively monitor prices of competitors Little menu costs to change prices swiftly and repeatedly to respond to price actions of competitors Increased availability of price and demand data Higher impact of competitor pricing on demand for own product offerings (Brynjolfsson and Smith 2000; Elmaghraby and Keskinocak 2003)
Offline	Offline brick-and-mortar store experience Travel and inconvenience costs High barriers for consumers to acquire information about other sellers’ prices Lower impact of competitor pricing on buying decision (Devaraj et al. 2002; Zhang et al. 2018a)	Offline brick-and-mortar store experience as lever to build more personal relationships with consumers Differentiation through loyalty Lower impact of competitor pricing on demand for own product offerings (Penz and Hogg 2011)

To date, a number of scholarly articles reviews various aspects of pricing under competition or online pricing (Boer 2015a; Chen and Chen 2015; Cheng 2017; Kopalle et al. 2009; Ratchford 2009; Vives 2001). Vives (2001) provides an overview of the history of pricing theory and its evolution from the early work of Bertrand (1883) who studied a duopoly with unconstrained capacity and identical products to Dudey (1992) who set the foundation for today’s dynamic pricing^4^ research with constrained capacities and a finite sales horizon. Ratchford (2009) reviews the influence of online markets on pricing strategies. Although he depicts factors shaping the competitive environment of online markets and compares online versus offline channels, he does not include competitive strategies specifically. This also holds for review papers on dynamic pricing which treat competition rather novercally (Boer 2015a; Gönsch et al. 2009). With emphasis on mobility barriers, multimarket contact and mutual forbearance, Cheng (2017) studies competition mechanisms across strategic groups. Kopalle et al. (2009) discuss competitive effects in retail focusing on different aspects such as manufacturer interaction and cross-channel competition. To the best of our knowledge, Chen and Chen (2015) are the only scholars who review existing competitive pricing research by classifying model characteristics along product uniqueness (identical vs. differentiated), type of customer (myopic vs. strategic), pricing policy (contingent vs. preannounced) and number of competitors (duopoly vs. oligopoly). However, competition is only one of three pricing problems they analyze forcing them to reduce scope and depth and to exclude online peculiarities. In addition, significant competitive pricing contributions were published since 2015 (chapter 2.2). Overall, given the limitations of previous reviews of the pricing literature makes revisiting the current state of research a worthwhile undertaking.

Most often, competitive pricing literature uses simplifying assumptions limiting the applicability of presented models. The simplifications are required to circumvent challenges like the *curse of dimensionality* (Harsha et al. 2019; Kastius and Schlosser 2022; Li et al. 2017; Schlosser and Boissier 2018), *endogeneity problems* (Cebollada et al. 2019; Chu et al. 2008; Fisher et al. 2018; Villas-Boas and Winer 1999), *uncertain information* (Adida and Perakis 2010; e.g., Bertsimas and Perakis 2006; Chung et al. 2012; Ferreira et al. 2016; Keskin and Zeevi 2017; Shugan 2002) and *simultaneity bias* (Li et al. 2017). As a consequence, early work on pricing strategies with competition was restricted to theoretical discussions (Caplin and Nalebuff 1991; Mizuno 2003; Perloff and Salop 1985). This holds especially true in combination with other practical circumstances such as capacity constraints, time-varying demand or a finite selling horizon (Gallego and Hu 2014).

Armstrong and Green (2007) find empirical evidence that competitive pricing, especially for the sake of gaining market share, harms profitability. Similarly, some researchers cursorily ascribe competitor-based pricing as a sign of a poor management because it signals a lack of capabilities to set prices independently (Larson 2019). Revenue management researchers therefore often assume that monopoly pricing models implicitly capture the dynamic effects of competition. The so-called *market response hypothesis* is the key rationale to neglect the effects of competition altogether (Phillips 2021; Talluri and van Ryzin 2004). According to this reasoning, competition does not have to be considered as all relevant effects are already included in historical sales data. However, this intuitive argument can be easily rebutted as Simon (1979) already showed that price elasticities change over time. Furthermore, Cooper et al. (2015) study the validity of the *market response hypothesis* and conclude that this monopolistic view is rarely adequate. Monopolistic pricing models can only be applied to stable markets with little time-varying demand and little expected competitive reactions.

Detrimental outcomes of ignoring competition in pricing strategies are shown by Anufriev et al. (2013), Bischi et al. (2004), Isler and Imhof (2008), Schinkel et al. (2002), and Tuinstra (2004). The negative effects are even more harmful in fierce competitive settings such as situations with a high number of competitors or price sensitive customers (van de Geer et al. 2019). Empirical evidence on the influence of competition on pricing decisions is provided by Richards and Hamilton (2006) who find that retailers compete on price and variety for market share. Li et al. (2017) observe that competition-based variables explained 30.2% of hotel price variations in New York—compared to 22.3% attributed to demand-side variables. Similarly, Hinterhuber (2008) assesses competitor-based pricing as a dominant strategy from a practical perspective. Li et al. (2008) argue that because of its relevance, competition should be considered in operational revenue management and not be treated stepmotherly as an abstract strategic constraint.

Although striving to simplify pricing models is desirable, researchers should thus not simply ignore effects of competition on price setting in a non-monopolistic (online) world. Blindly pegging pricing strategies to competitors or undercutting competitors to gain market share may favor detrimental price wars and not profit-maximizing market structures. Nevertheless, no significant market player can operate isolated on online markets—decisions made always affect competing firms and consumer demand (Chiang et al. 2007). In such dynamic markets (chapter 3.3), competition must be considered with time-varying attributes (Schlosser et al. 2016).

Against this background, we suggest a conceptual framework to structure research covering competitive online pricing. It can serve scholars as a map to direct future research on the one hand and provide practicing managers with a guide to locate relevant pricing contributions on the other hand. Although the framework can be applied to a variety of markets with competitive dynamics, we concentrate our review on research covering B2C online goods retail markets. Thus, related research with a focus on auction pricing, multichannel peculiarities, behavioral pricing and multi-dimensional pricing approaches such as Everything-as-a-Service (XaaS) or bundle pricing is only assessed when findings are crucial to the competition-related discussion. In the remainder, we proceed as follows. The next chapter provides a descriptive overview of the competitive pricing literature for the subsequent discussion. Chapter 3 puts the identified literature into the perspective of online retail markets considering product and environmental characteristics. Section 4 concludes with practical implications and directions for future research.

## Overview of competitive pricing research

Initially, properties of the reviewed literature are briefly summarized. Besides (a) the journal representation, (b) the historical development of online market considerations and (c) research domains, we classify research according to (d) the geographical and industry context as well as (e) research design and empirical foundation.

We identified relevant references through a semi-structured multi-pronged search strategy. Following Tranfield et al. (2003), we firstly screened the literature reviews mentioned in chapter 1 to obtain an overview of existing research streams. Second, we created a set of potentially relevant contributions by searching multiple keywords in the journal databases EBSCO, Scopus and Web of Science (c.f. Baloglu and Assante 1999).^5^ Third, high-impact journals (see Appendix 1) in the academic fields economics, marketing management, and operations were screened. With focus on highly cited (> 10 citations in Scopus), recent (published later than 2000) research, we identified an initial sample of 996 unique papers. Fourth, we studied the abstract and skimmed the text of all papers for relevance to competitive online pricing, reducing our initial set to 174 papers. Fifth, we screened the references of the papers and identified literature cited which we not already included in our set. Sixth, especially for research areas with limited coverage in peer-reviewed journals, we uncovered *gray literature* through searches with Google Scholar. As a result, this study concentrates on papers published between 2000 and 2022 and only sparsely utilizes literature from the pre-internet era. The final sample of the papers with relevance to competitive B2C online pricing encompasses 132 entries. A complete list of the papers reviewed in great depth is provided in Appendix 2. 94% are peer-reviewed articles. Book chapters, conference papers and preprint/working papers each account for 2%.

### Journal representation

Competitive pricing literature is widely dispersed over a broad range of journals as roughly half of the articles considered are from journals with less than three articles in our review. Notably, journals with a higher density of competitive pricing contributions are from the fields of operations, economics or are interdisciplinary. Table 2 reports the distribution of articles among the journals with the highest representation. In addition, it provides the considered articles subject to a content analysis in chapter 3.

**Table 2:** Overview of most significant journals for competitive pricing research by number of publications and articles from these journals

Journal^a^	# of articles	Articles reviewed
Management Science	18	Abhishek et al. (2016), Aksoy-Pierson et al. (2013), Anand and Girotra (2007), Bernstein and Federgruen (2005), Cachon and Harker (2002), Campbell et al. (2005), Chioveanu and Zhou (2013), Gallego and Hu (2014), Jerath et al. (2010), Levin et al. (2009) Li et al. (2017), Liu and Zhang (2013), Martínez-de-Albéniz and Talluri (2011), Miklós-Thal and Tucker (2019), Netessine and Shumsky (2005), Olivares and Cachon (2009), van Mieghem and Dada (1999), Viswanathan (2005)
Production and Operations Management	10	Balakrishnan et al. (2014), Besbes and Sauré (2016), Dong et al. (2019), Kachani et al. (2010), Lin et al. (2014), Mookherjee and Friesz (2008), Ryan et al. (2012), Sun and Gilbert (2019), Wang and Hu (2014), Yang and Xia (2013)
Marketing Science	9	Balasubramanian (1998), Blattberg and Wisniewski (1989), Caillaud and Nijs (2014), Lal and Sarvary (1999), Moorthy (1988), Netzer et al. (2012), Ringel and Skiera (2016), Thomadsen (2007), Villas-Boas (2004)
European Journal of Operational Research	7	Bernstein et al. (2008), Dasci and Karakul (2009), Geng and Mallik (2007) Lin and Sibdari (2009), Matsubayashi and Yamada (2008), Wang et al. (2017), Zhao et al. (2017)
Operations Research	6	Adida and Perakis (2010), Bernstein and Federgruen (2004), Cooper et al. (2015), Federgruen and Hu (2015), Gallego and Wang (2014), Lippman and McCardle (1997)
RAND Journal of Economics	5	Chen et al. (2018), Chioveanu (2012), Dana and Petruzzi (2001), Farias et al. (2012), Villas-Boas (1999)
American Economic Review	4	Calvano et al. (2020), Dinerstein et al. (2018), Salop (1976), Stiglitz (1979)
Journal of Revenue and Pricing Management	4	Isler and Imhof (2008), Kastius and Schlosser (2022), Schlosser and Richly (2019), van de Geer et al. (2019)
Econometrica	3	Caplin and Nalebuff (1991), Maskin and Tirole (1988), Weintraub et al. (2008)
Journal of Economics and Management Strategy	3	Chen (1997), Loginova (2021), Villas-Boas (2006)
Journal of Retailing	3	Choi (1996), Dickson and Urbany (1994), Fay (2008)

^a^Journals with more than 2 contributions account for 56.8% of articles subjected to an in-depth content analysis

### Online pricing contributions over time

**Fig. 1:**
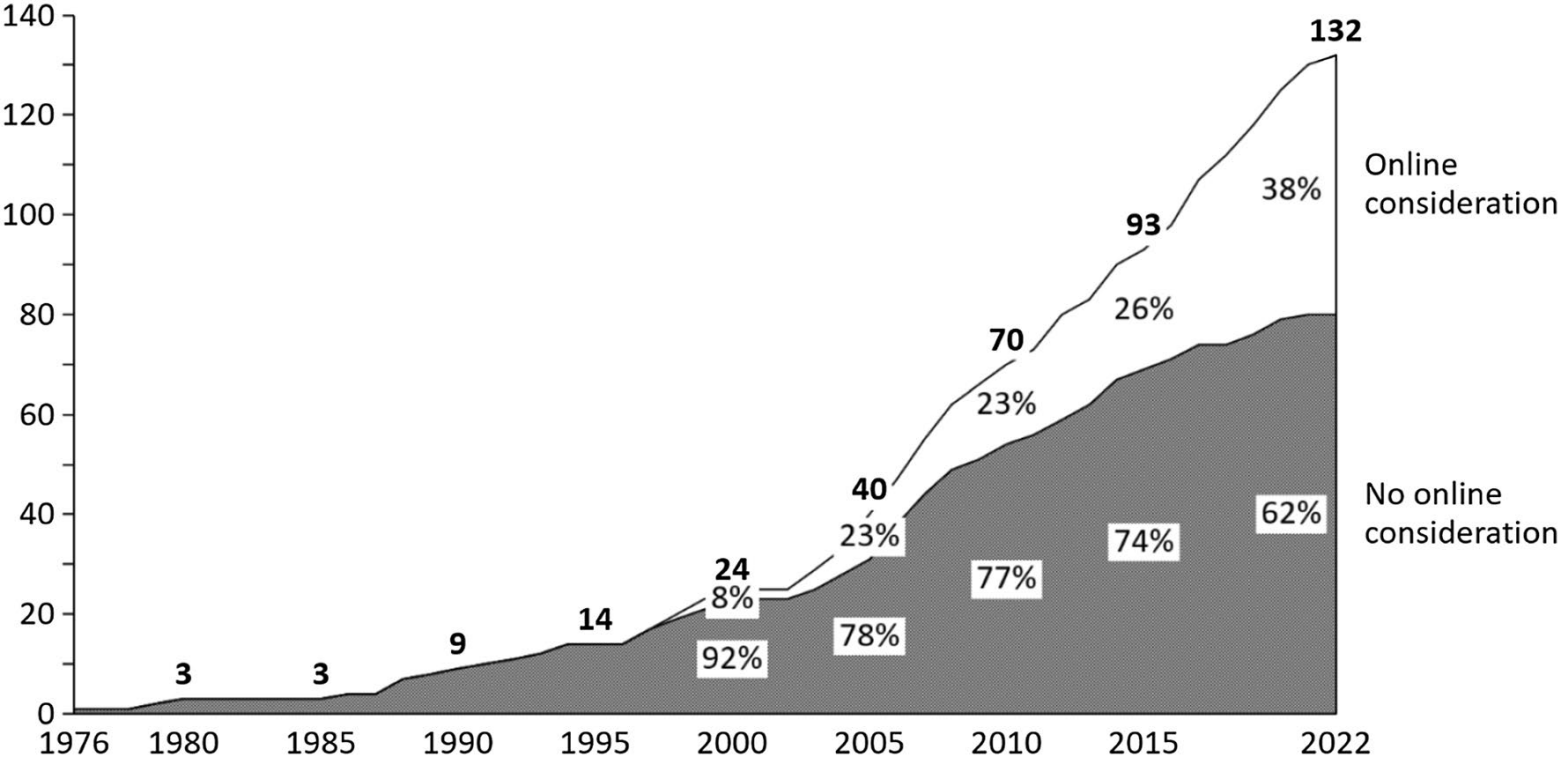
Competitive pricing literature and its consideration of online peculiarities accumulated by year

Between 1976 and the end of the second millennium, the number of papers on competitive pricing in an internet context is naturally limited (Fig. 1). Parallel to the dissemination of online use among residential households, interest of researchers in online pricing in a competitive environment started to take off. 71.8% of the papers published from 2015 to 2022 consider online settings specifically. The corresponding statistic from 2000 to 2005 amounts to 43.8%.

### Development of research domains

Competitive pricing literature typically can be assigned to one of the following research domains:

The *economics domain* takes a market perspective across individual firms. It elaborates on the existence and uniqueness of competitive equilibria also including all subjects regarding econometrics.

The *marketing management domain* analyzes competitive pricing problems from the perspective of a single firm with a focus on customer reactions to pricing decisions. It includes all subjects linked to marketing, strategy, business, international, technology, innovation, and general management.

The *operations domain* considers quantitative pricing solutions for, among others, quantity planning, choice of distribution channels, and detection of algorithm driven price collusion. It includes all subjects regarding computer science, industrial and manufacturing engineering, and mathematics.

**Fig. 2:**
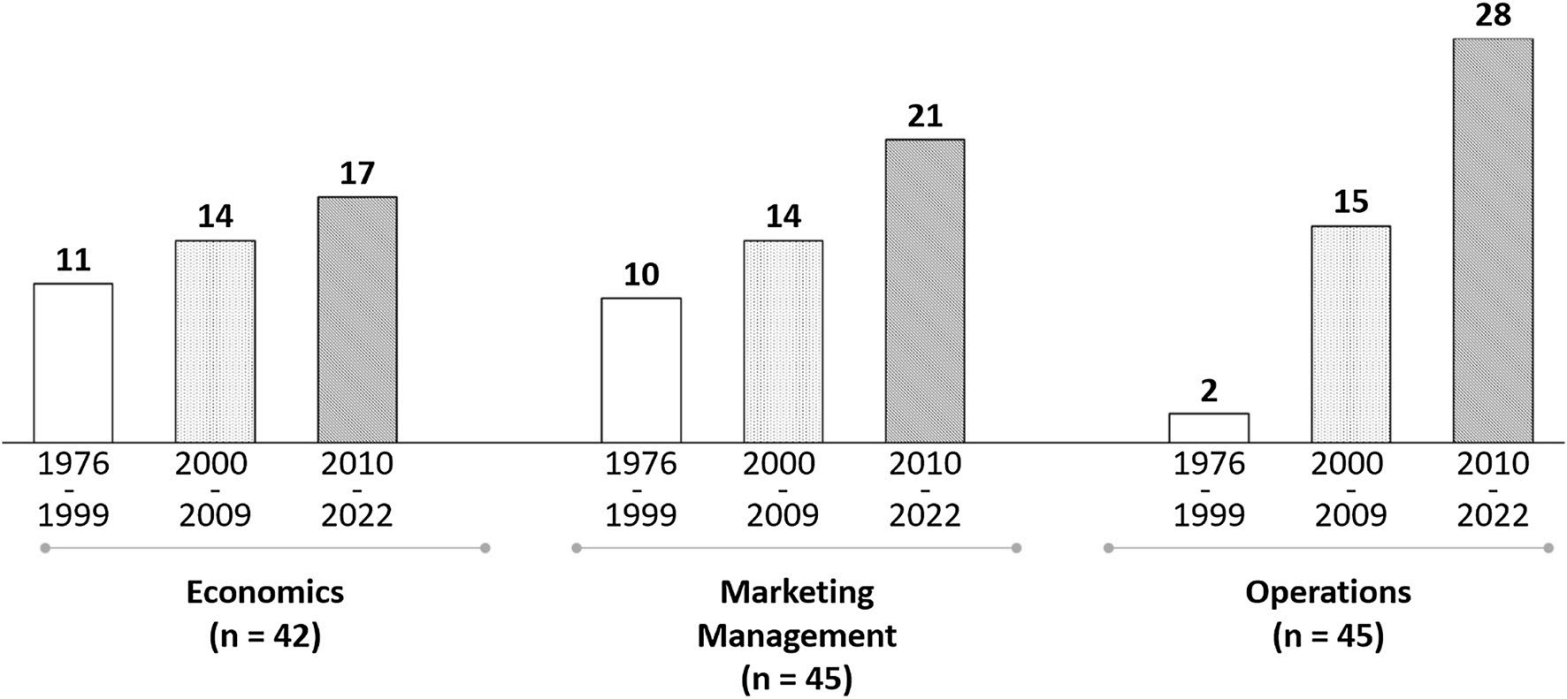
Distribution of competitive pricing literature over research domain and time interval

Separating the last 47 years of competitive pricing research into three intervals, all reviewed papers are assigned to their most affiliated research domain. Although the domains are similarly represented in our review (see Fig. 2), we see differences in their temporal change. Whereas rather theoretical economic subjects are covered relatively constant over time, more practice-oriented marketing management and operations subjects gained momentum since 2000. This suggests a shift from model conceptualization toward applicable research, frequently based on empirical data.

### Geographical and industry context

**Fig. 3:**
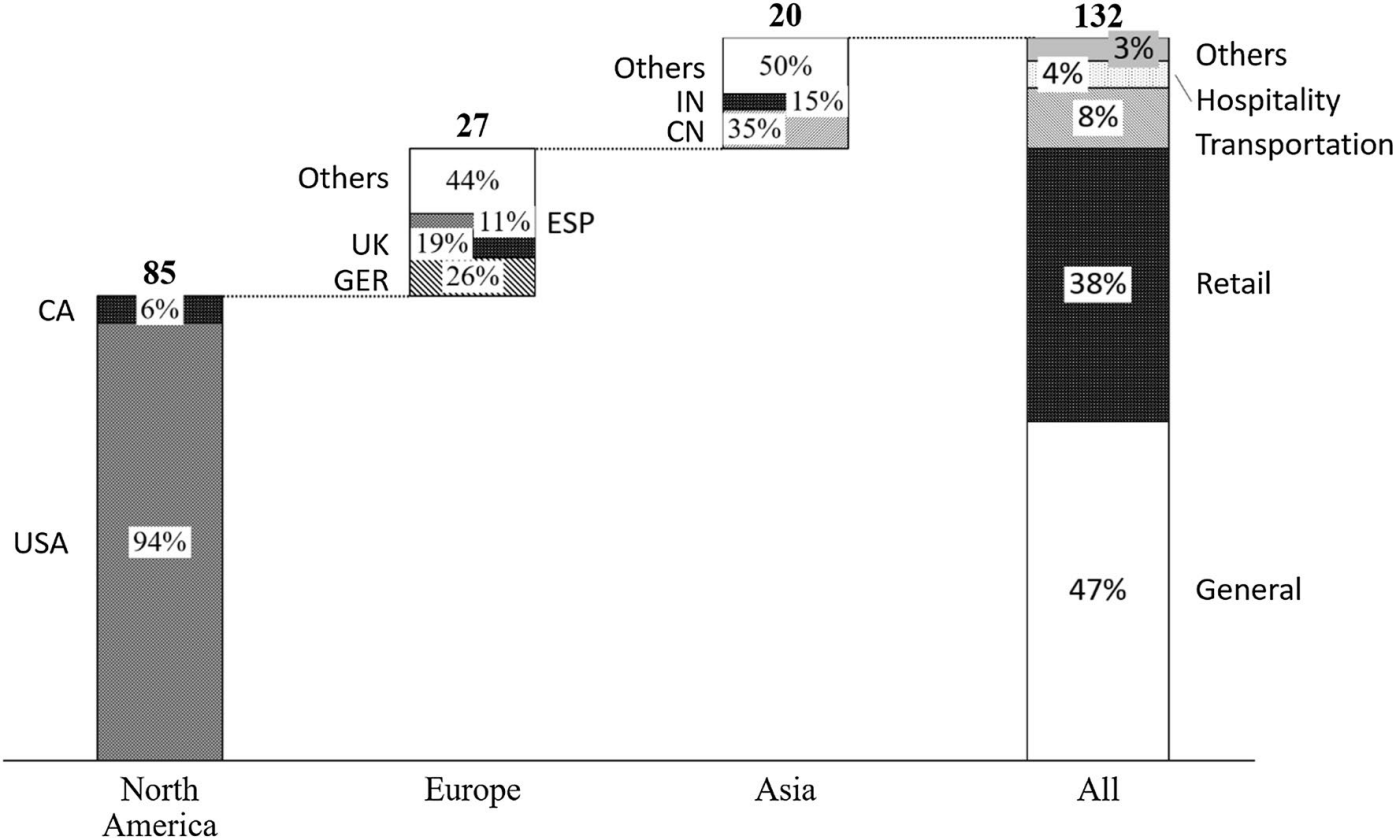
Competitive pricing research by focal industry and location of lead authors’ institution

As the origin of revenue management lies in transportation and hospitality optimization problems, one could expect that competitive pricing research also originates in these dynamic sectors. However, our analysis reveals a different picture: Almost half of the papers in our review do not concentrate on a specific industry. Besides, most industry-specific competitive pricing articles focus on retail, with 38% concentrating on the retail industry versus 8% and 4% on transportation and hospitality, respectively (see Fig. 3). This supports our proposition in chapter 1 that effects of competition on industry-specific pricing are particularly relevant for online markets.

Competitive pricing literature is predominantly driven by researchers employed by U.S. institutions (60%). The remaining 40% consist of Europe (19%), Asia (17%) and Canada (4%).

### Research approach

A lack of empirical testing is an issue that hampers competitive pricing research. Liozu (2015) reported that only 15% of general pricing literature include empirical data. For competitive pricing, the situation appears even more aggravated. In addition to parameters such as price elasticities and stock levels of the company under study, comprehensive, real-time information of other market participants is crucial to add practical value.

For instance, to solve a simple Bertrand equilibrium,^6^ full information of all competitors is needed, which is rarely available in real-life settings. Therefore, many problems covered in the literature are of a theoretical nature. In accordance with Liozu (2015), we find that only 18% of reviewed articles use empirical evidence to validate hypotheses. An additional 23% strive to ameliorate this shortage through simulation data and numerical examples. The remaining 59% fail to bring any empirical evidence or numerical examples.

**Fig. 4:**
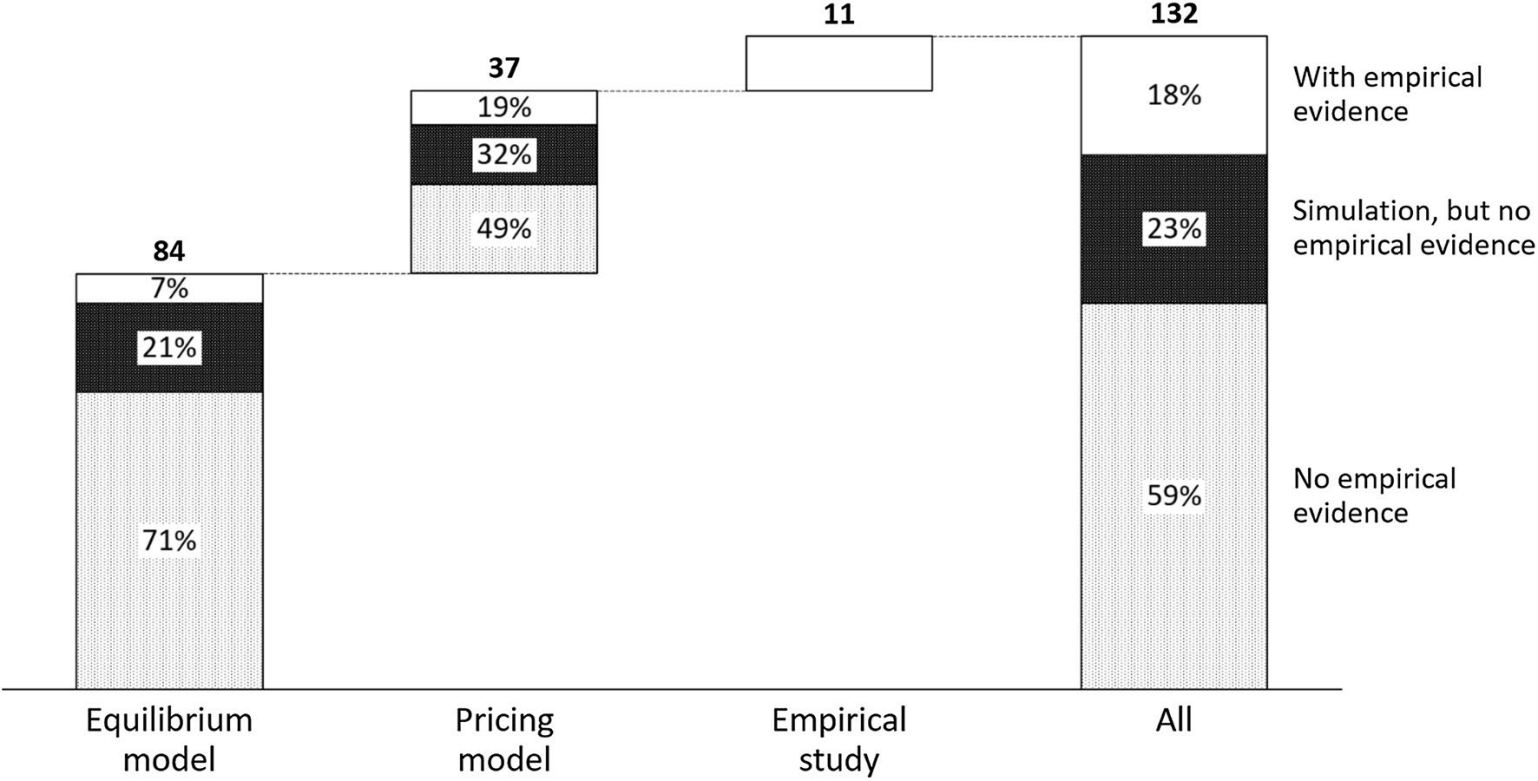
Competitive pricing research by research design and empirical validation

As can be taken from Fig. 4, missing empirical support is particularly prevalent for equilibrium models which use empirical data in only 7% of all papers.

## Competitive pricing on online markets

In this chapter, we assess the applicability of competitive pricing work to online markets. Typical characteristics of competitive B2C pricing models were derived from literature described in chapter 2. Competitive pricing literature can be classified along four characteristics depicted in Table 3 that form the market environment in which firms compete for consumer demand.

**Table 3:** Key questions and classification parameters of competitive model characteristics

Model characteristic	Key question	Attributes
Product similarity	How similar are product characteristics across firms?	Identical / quality differentiated goods
Product durability	Are products to be considered durable and how can this affect pricing objectives?	Perishable / durable goods
Time dependence	Should competitive pricing problems be considered dynamically?	Time-dependent / time-independent setting
Market structure	How far does the market structure of interacting firms influence the nature of price competition?	Monopolistic / duopolistic / oligopolistic / perfect competition

In the remainder of chapter 3, we discuss the four key questions in more depth and elaborate on their applicability to online retail markets.

### Product similarity

In general, products in competitive pricing models are either identical (homogeneous) or differentiated by at least one quality parameter (heterogeneous). In case of homogeneous products, pricing is the only purchase decision variable—a perfectly competitive setting (Chen and Chen 2015). However, many firms strive to differentiate their products as this shifts the focus from the price as competitive lever to other product-related features (Afeche et al. 2011; Boyd and Bilegan 2003; Thomadsen 2007). According to Lancaster (1979), there are two types of product differentiation: vertical and horizontal differentiation. Vertical differentiation^7^ encompasses all product distinctions which are objectively measurable and quantifiable regarding their quality level. Horizontal differentiation^8^ can manifest in many variants and includes all product-related aspects which cannot be quantified according to their quality levels.^9^ A key difference in the modeling of substitutable yet differentiated versus identical goods is that customers have heterogenous preferences among products.^10^ A recent stream of literature approaches unknown differentiation criteria by assessing online consumer-generated content (DeSarbo and Grewal 2007; Lee and Bradlow 2011; Netzer et al. 2012; Ringel and Skiera 2016; Won et al. 2022).

Besides the chosen price level, Cachon and Harker (2002) argue that firms compete with the operational performance level offered and perceived, i.e., service level in online retail, to differentiate an otherwise homogenous offering. In situations, where resellers with comparable service and shipping policies offer similar products, price is a major decision variable for potential buyers (Yang et al. 2020). Often, e-tailers do not possess the right to exclusively distribute a certain product. For example, Samsung’s Galaxy S21 5G was offered by 69 resellers on the German price comparison website Idealo.de.^11^ As some products in e-tail can be differentiated and others cannot, both identical and differentiated product research have their *raison d’être* for competitive online pricing.

Most competitive pricing models only address the effects of single-product settings. This simplification is reasonable if there is no interdependence between products of an e-tailer (Gönsch et al. 2009). Taking up on the smartphone example, the prices of close substitutes, such as Huawei’s P30 Pro, nonetheless have an impact on the demand of Samsung’s Galaxy S21 5G. To further extent product differentiation, price models have to incorporate multi-product pricing problems in non-cooperative settings (Chen and Chen 2015). Such models have to account not only for demand impact of directly competing products but also for synergies, cannibalization/substitution effects of (own) differentiated goods. Although there is a recent research stream regarding product assortment (Besbes and Sauré 2016; Federgruen and Hu 2015; Heese and Martínez-de-Albéniz 2018; Nip et al. 2020; Sun and Gilbert 2019), multi-product work is still underdeveloped. Thus, competitive multi-product pricing constitutes an area which should be addressed in future research.

### Product durability

The durability of products is an important feature to differentiate between competitive pricing model types. Durable (non-perishable) products do not have an expiration date, for example consumer durables such as household appliances. Perishable products can only be sold for a limited time interval and have a finite sales horizon. After expiration date, unused capacity is lost or significantly devalued to a salvage value.^12^ Combined with limited capacities, the firm objective is thus most often to maximize turnover under capacity constraints and finite sales horizon (Gallego and van Ryzin 1997; McGill and van Ryzin 1999; Weatherford and Bodily 1992).

Perishability can be of relevance for products with seasonality effects or short product life cycles (i.e., finite selling horizon) such as apparel, food groceries or winter sports equipment. This is especially relevant because online retailers of perishable products are severely restricted in their shipment, return handle policies and supply chain length (Cattani et al. 2007). Sellers cannot replenish their inventory after the planning phase and cannot retain goods for future sales periods (Perakis and Sood 2006). Some products like apparel—albeit reducing in value after a selling season—still have a certain salvage value and can be sold at reduced prices (Anand and Girotra 2007).

It depends on the type of product to decide whether perishability should be included in competitive pricing models. There is a fundamental distinction in the underlying optimization objective for models with or without perishability. Whereas models with perishable products tend to focus on revenue maximization over a definite *short-term* time horizon, models with durable products tend to focus on profit maximization over an indefinite or at least *long-term* time horizon by balancing current revenues of existing and future revenues of new customers. To account for this trade-off, models with durable products need to discount future cash flows incorporating time value of money, stock-keeping, opportunity and other costs related to prolonged sales (Farias et al. 2012). To conclude, perishability cannot be treated as an extension to durable models but rather as a separate class of pricing models. Depending on the product and/or setting in focus, both are relevant for online retailing. Further research could investigate the performance of models with and without consideration of perishability in various (online) settings to determine when it is appropriate to use which class of pricing models. Also, an interesting field of future studies arises around the question which instruments (e.g., service differentiations or price diffusion) are used by online retailers to differentiate otherwise homogeneous offerings.

### Time dependence

A key differentiator of competitive pricing models is the consideration of either a static (time-independent) setup with definite equilibrium or a dynamic (time-dependent) constellation with changing environmental factors and equilibria. Albeit static pricing models have no time component, many consist of multiple stages to investigate the interplay of different factors.^13^ In contrast, dynamic models allow for varying competitive (re-)actions over time.^14^ Within the latter category, there are models with a finite (Afeche et al. 2011; Levin et al. 2008; Liu and Zhang 2013; Yang and Xia 2013) and an infinite (Anderson and Kumar 2007; Li et al. 2017; Schlosser and Richly 2019; Villas-Boas and Winer 1999; Weintraub et al. 2008) time horizon.

Historically, competitive pricing models assumed fixed prices over the considered time horizon. Limited computational power made it impossible to appropriately estimate models dynamically due to dimensionality issues (Schlosser and Boissier 2018). A lack of reliable demand information, high menu and investment costs to implement dynamic approaches were additional reasons why pricing models remained inherently static without incorporating changing competitive responses (Ferreira et al. 2016). The focus in retail has conventionally rather been on long-term profit optimization and to a lesser degree on dynamically changing price optimizations (Elmaghraby and Keskinocak 2003).

The literature disagrees on whether firms should opt for static or dynamic pricing strategies. A static environment allows to simplify and concentrate on a specific topic such as equilibrium discussions. For instance, Lal and Rao (1997) study success factors of everyday low pricing and derive conditions for a perfect Nash equilibrium between an everyday low price retailer and a retailer with promotional pricing. With Zara as an example for a company with a successful static pricing strategy, Liu and Zhang (2013) argue that with the presence of strategic customers who prolong sales in anticipation of price decreases, firms might even be better off to deploy static over dynamic price setting processes. Studying the time-variant pricing plans in electricity markets, Schlereth et al. (2018) suggest that consumers might prefer static over dynamic pricing because of factors like choice confusion, lack of trust in price fairness, perceived economical risk or perceived additional effort. Further support for a static pricing strategy is found in Cachon and Feldman (2010) and Hall et al. (2009).

Nevertheless, to generalize that static should strictly be preferred over dynamic pricing models could be short-sighted. Firms cannot generally infer future behavior of competitors from past observations to assess how competitive (re-) actions may influence the optimal pricing policy (Boer 2015b). Corresponding to the surge of revenue management systems in the airline industry during the 70s and 80s, increased price and demand transparency, low menu costs and an abundance of decision support software created fierce competition among online retailers (Fisher et al. 2018). Taking up on the above mentioned example by Liu and Zhang (2013), Caro and Gallien (2012) show that even Zara does not solely rely on static pricing. They supported Zara’s pricing team in designing and implementing a dynamic clearance pricing optimization system—to generate a competitive advantage in addition to the fast-fashion retail model Zara mainly pursues (Caro 2012). Zhang et al. (2017) discuss various duopoly pricing models with static and dynamic pricing under advertising. They find that market surplus is highest when one firm prices dynamically, profiting from the static behavior of the other. Chung et al. (2012) provide numerical evidence that a dynamic pricing model with an appropriately specified demand estimation always outperforms static pricing strategies—also in settings with incomplete information. Xu and Hopp (2006) show that dynamic pricing outperforms preannounced pricing, especially with effective inventory management and elastic demand. Further support for advantages of dynamic pricing can be found by Popescu (2015), Wang and Sun (2019), and Zhang et al. (2018b). Empirical evidence of the negative consequences of sticking to a static strategy in a changing environment is found in the cases of Nokia, Kodak, and Xerox.

While some scholars distinguish between discrete and continuous dynamic pricing systems (Vinod 2020), we suggest to classify dynamic pricing models according to their level of sophistication into two evolutionary stages: the (in e-commerce widely applied) manual rule-based pricing approach and the data-driven algorithmic optimization approach (Popescu 2015; Le Chen et al. 2016).^15^ For the rule-based approach, “if-then-else rules” are defined and updated manually.^16^ However, the mere number of stock-keeping units (SKUs) in today’s retailer offerings aggravate the initial setup and handling of rule-based pricing and make real-time adjustments unmanageable (Schlosser and Boissier 2018). In addition, rule-based approaches are rather subjective than sufficiently data-driven. Faced with a large range of SKUs, competitor responses and heterogeneous demand elasticities, canceling out the human decision-making process on an operational level is the next evolutionary step for competitive pricing systems (Calvano et al. 2020). Data-driven algorithmic pricing strategies use observable market^17^ data to predict sales probabilities based on consumer demand and competitive responses (Schlosser and Richly 2019).

As online marketplaces benefit from an increased number of retailers on their platforms, they typically support sellers to establish automated dynamic pricing systems (Kachani et al. 2010).^18^ However, Schlosser and Richly (2019) claim that current dynamic pricing systems are not able to deal with the complexity of competitor-based pricing and therefore most often ignore competition altogether or solely rely on manually adjusted rule-based mechanics. Challenges include the indefinite spectrum of changing competitor strategies, asymmetric access to competitor knowledge, a large solution space under limited information and the black-box character of dynamic systems, which exacerbates an intervention in case of a pricing system malfunction. Besides, researchers did not yet identify an algorithm which consistently outperforms other methodologies in competitive situations. Instead, it depends on the specific setting and other competitors’ pricing behavior to assess which pricing algorithm is optimal (van de Geer et al. 2019) exacerbating the application of such systems.

Reflecting the literature findings for both static and dynamic pricing strategies, we conclude that pricing managers should develop dynamic pricing models in most e-commerce situations. As long as demand and competitor price responses vary over time on online markets, dynamic models are naturally superior to time-independent approaches. Static models on the other hand are only appropriate in market constellations with little time-varying demand and competitor behavior. As static research can be expected to remain a vivid field of literature, further research with regard to the transferability of static models to dynamic settings is desirable. In addition, more research is needed that helps to better understand the implications of widely applied rule-based dynamic pricing methods and their transition toward algorithmic approaches (Boer 2015a; van de Geer et al. 2019; Kastius and Schlosser 2022; Könönen 2006).

### Market structure

The market structure describes the number of competing firms such as duopoly or oligopoly in a demand setting with an indefinite number of consumers. 60% of the reviewed papers studied duopolies, 49% oligopolies, 7% monopolistic competition, and 3% perfect competition.^19^

Especially for research in the economics stream, many papers assume a perfectly competitive market. Pricing research with perfectly competitive markets (e.g., van Mieghem and Dada 1999 or Yang and Xia 2013) is likely to be of very limited value to online retailers. Building on the notion of Diamond (1971), Salop (1976) argues that if customers have positive information gathering costs, no perfect competition can occur as firms have room to slightly increase prices without losing demand. Christen (2005) found evidence that even with strong competition and low information costs, cost uncertainty could decrease the detrimental effect of competition for sellers and could increase prices above Bertrand levels. Similarly, Bryant (1980) showed that perfect competition is not possible in a market with uncertain demand, even if the number of firms is large and customers have no search costs. Rather, price dispersion reflects uncertain demand (Borenstein and Rose Nancy L. 1994; Cavallo 2018; Clemons et al. 2002; Obermeyer et al. 2013; Wang et al. 2021). Israeli et al. (2022) empirically show that the market power of individual firms does not only depend on the number and intensity of competitors but also on the firm’s ability to adjust prices in response to varying inventory levels of product substitutes, especially with low consumer search costs. This is of relevance for e-commerce as e-tailers could exploit this dependence by incorporating competitors’ stock levels into pricing decisions (Fisher et al. 2018).

Some papers discuss (quasi) monopolistic competition (e.g., Xu and Hopp 2006) in which small firms charge the (higher) monopoly price rather than the (lower) competitive price. From an empirical study in the U.S. airline industry, Chen (2018) concludes that, as firms can price discriminate late-arriving consumers, competition is softened, profits are increased, and the only single-price equilibrium could be at the monopoly price. This supports Lal and Sarvary (1999) who show that online retailers enjoy a certain amount of monopoly power in cases where buyers cannot switch suppliers for repeated purchases (e.g., technical incompatibility reasons). In such cases, switching costs could increase online prices (Chen and Riordan 2008). However, this contradicts Deck and Gu (2012) who empirically show that, although the distribution of buyer values of competing products might theoretically lead to higher prices through competition, intensity of competition rarely allows for an occurrence of this phenomenon in e-tail settings.

Although duopoly settings can serve to assess the relevant strength of pricing strategies, which is not directly possible for oligopoly markets due to the *curse of dimensionality* (Kastius and Schlosser 2022), they cannot be transferred to more competitive environments (van de Geer et al. 2019). In online retail, a duopoly market structure is a rare exemption. Like for perfectly competitive markets, findings of duopoly research must be carefully assessed in terms of their applicability to online retail oligopolies.

Bresnahan and Reiss (1991) found empirical evidence that markets with an increasing number of dealers have lower prices than in less competitive market structures such as monopolies or duopolies. Although applicable to many online retail markets, where retailers face dozens, if not hundreds of thousands of competitors (Schlosser and Boissier 2018), few research attention is currently given toward a structure with a large number of competitors in an imperfect market (cf. Li et al. 2017). A way to assess the current competitive structure of markets is the utilization of online consumer-generated content such as forum entries (Netzer et al. 2012; Won et al. 2022) or clickstream data (Ringel and Skiera 2016) and actual sales data (Kim et al. 2011).

In many countries with well-developed B2C online markets, one or few major retailers dominate on an oligopolistic market. For example, the top three online retailers in the United States accounted for over 50% of the revenue generated on the national e-commerce market in 2021.^20^ Due to lower locational limitations in conjunction with substantial economies of scale and scope, online markets tend to become more concentrated than their offline counterparts (Borsenberger 2015). Although one could expect that increased market transparency leads to a higher intensity of competition (Cao and Gruca 2003), limiting the market power of established firms and leaving growth potential for smaller firms (Zhao et al. 2017), it appears reasonable to predict that most online markets will ultimately resemble an oligopoly setting with a with a relatively small number of players—enabling increased tacit pricing algorithm collusion in the future (Calvano et al. 2020). With few exceptions (e.g., Noel 2007), there is little research (Brown and Goolsbee 2002; Wang et al. 2021; Cavallo 2018) exploring what type of competitor-based pricing strategies are used and what competitive dynamics are found on e-tail markets. Thus, more research is needed to investigate the current state of market structure and intensity of competition in today’s e-commerce markets as drivers of the selection and the outcomes of pricing approaches.

## Implications and directions

We contribute to the literature by providing an interdisciplinary review of competitive online retail research. Competitive pricing problems can most often be assigned to one of the academic fields of economics, marketing management or operations. In a first step, this review offered a descriptive portrayal of the relevant literature. Motivated by practical issues and common features in competitive pricing research, we then structured competitive pricing contributions along four properties of pricing models. First, do firms compete with identical or quality differentiated products? Second, are products to be considered as perishable or durable goods? Third, is the market setting to be regarded time-independent or not? Fourth, which market structure prevails on e-tail markets? The framework is derived from an analysis of pricing research not exclusively restricted to online retail settings. Therefore, it could be extended to other online or offline markets, with little loss of generalizability.

We focused on e-tail markets because the relevance of competition for pricing strategies is disproportionally higher in such environments. On e-tail online markets, products are rarely offered exclusively so that the likelihood of substitutive competition is high. Nevertheless, products can be differentiated through other factors than prices such as generous shipping, customer retention (e.g., loyalty reward programs) or return and issue handling policies. With a look on product similarities, accounting for product interdependencies and multi-product situations are important improvements of prevailing pricing models. Second, pricing models with both a focus on perishable and/or durable products are relevant on e-tail markets. However, further research is needed exploring which of the respective perishability considerations are appropriate for different settings. Third, we conclude that, albeit time-independent static models may occasionally serve to simplify pricing issues, dynamic models outperform their static counterparts in constantly changing market environments such as in e-commerce. Fourth, we show that in most practical settings, online markets resemble either an oligopolistic market structure or a structure with many firms under imperfect competition. Thus, future research should consider these two “real” competitive settings instead of further looking at simplifying market structures such as monopolistic or duopolistic competition. This should ease a transfer of theoretical insights into practical applications. To sum, firms should be able to improve their competitive position by developing a profit optimizing dynamic pricing strategy for identical products in an oligopolistic setting with a varying number and relevance of competitors.

Due to space limitations, we had to focus on competitive pricing model characteristics related to four overall product and market attributes. Thus, more work is needed on other characteristics of competitive pricing models, particularly firm- and consumer-related characteristics. Firm-related characteristics encompass various additional properties of interacting firms (e.g., similarity or capacity constraints). Similarly, consumer-related characteristics entail further properties of interacting buyers (e.g., certainty, discreteness, sophistication, and homogeneity of demand).

In the selection process of literature, this study only considered papers in peer-reviewed journals and conference proceedings in English. Subsequent research could complement our findings by including industry-funded, unpublished and non-peer-reviewed articles, also in other languages. In addition, we do not claim that our research captures all competitive pricing publications of the considered field. As our study spans almost 50 years of a frequently discussed topic in the domains of economics, marketing management, and operations, we had to constrain the scope to the most influential work. Although we mutually evaluated our selection decisions and consulted outside peers for validation and further input, we cannot eliminate the element of subjectivity. Consequently, other authors could have selected slightly different papers. However, this shortcoming is unlikely to significantly affect our results as our literature selection was derived from a broad array of competitive pricing research and would therefore be only marginally influenced by a few omitted articles.

## Appendix 1

See Table 4.

**Table 4:** Selection of systematically reviewed journals and classification of scientific relevance

Journal name	Percentile in discipline	SJR 2020^a^	VHB-JQ3 rating 2021^b^
Interdisciplinary journals			
Journal of Marketing	Q1	7.8	A +
Marketing Science	Q1	5.9	A +
Management Science	Q1	5.0	A +
Information Systems Research	Q1	3.5	A +
Quantitative Marketing and Economics	Q1	2.1	B
Decision Sciences	Q1	1.2	B
Journal of the Operational Research Society	Q1	0.8	B
Journal of Revenue and Pricing Management	Q3	0.3	C

Economics journals			
American Economic Review	Q1	17.0	A +
Econometrica	Q1	16.7	A +
RAND Journal of Economics	Q1	3.7	A
International Journal of Production Economics	Q1	2.4	B
Journal of Economics and Management Strategy	Q1	1.7	A
Journal of Industrial Economics	Q1	0.9	A

Marketing management journals			
Journal of Service Research	Q1	4.4	A
Journal of Retailing	Q1	3.2	A
Omega	Q1	2.5	B
International Journal of Electronic Commerce	Q1	1.2	B
Journal of Product and Brand Management	Q1	1.0	C

Operations journals			
Manufacturing and Service Operations Management	Q1	7.4	A
Operations Research	Q1	3.8	A +
Production and Operations Management	Q1	3.3	A
IEEE Transactions on Industrial Informatics	Q1	2.5	A
Mathematical Programming	Q1	2.4	A
European Journal of Operational Research	Q1	2.2	A
Computers and Operations Research	Q1	1.5	B
Central European Journal of Operations Research	Q2	0.7	C

^a^SJR: SCImago Journal Rank

^b^The Association of University Teachers of Business Administration e.V. (VHB) is made up of over 2,800 business administration (BA) academics. Founded in 1921, VHB is the leading scientific institution of BA in German-speaking countries. JOURQUAL3 is a rating of journals relevant to business administration based on the judgments of VHB members. The rating categories are: A + : outstanding and world leading scientific journals in BA (= 3.4%); A: leading scientific BA journals (11.1%); B: important and respected scientific BA journals (33.3%); C: recognized scientific business journals (41.9%); D: scientific business administration journals (9.1%). The remaining 1.2% consist of in between categories

## Appendix 2

See Table 5.

**Table 5:** List of papers reviewed in-depth by research design

Research design	Empirical data	Online specified	Industry	Product Similarity	Durability	Time dependence	Time horizon	Market form	Author (Year)
Equilibrium model	Yes	Yes	Retail	Identical	Durable	Independent	1-stage	Duopolistic	Ba et al. (2012)
				Differentiated	Durable	Dependent	Finite horizon	Oligopolistic	Dinerstein et al. (2018)
			Transportation	Differentiated	Perishable	Dependent	Finite horizon	Duopolistic	Chen (2018)
								Oligopolistic	Chen (2018)
		No	Hospitality	Differentiated	Durable	Independent	2-stage	Duopolistic	Thomadsen (2007)
			Retail	Both	Durable	Dependent	3-stage	Oligopolistic	Dana and Williams (2020)
				Multi	Perishable	Dependent	Finite horizon	Oligopolistic	Richards and Hamilton (2006)
	Simulation	Yes	General	Identical	Durable	Both	Finite horizon	Duopolistic	Zhang et al. (2017)
						Dependent	Finite horizon	Duopolistic	Kastius and Schlosser (2022)
									Kutschinski et al. (2003)
								Oligopolistic	Kastius and Schlosser (2022)
									Kutschinski et al. (2003)
						Independent	1-stage	Duopolistic	Li et al. (2019)
					Perishable	Dependent	Finite horizon	Monopolistic	Lin and Sibdari (2009)
								Oligopolistic	Lin and Sibdari (2009)
				Differentiated	Durable	Dependent	Infinite horizon	Oligopolistic	Calvano et al. (2020)
			Retail	Identical	Durable	Both	Infinite horizon	Duopolistic	Campbell et al (2005)
			Social Media	Differentiated	Durable	Independent	2-stage	Duopolistic	Chen et al. (2018)
		No	General	Identical	Durable	Dependent	Infinite horizon	Duopolistic	Könönen (2006)
								Oligopolistic	Farias et al. (2012)
						Independent	2-stage	Duopolistic	Van Mieghem and Dada (1999)
								Oligopolistic	Van Mieghem and Dada (1999)
								Perfect comp	Van Mieghem and Dada (1999)
					Perishable	Both	2-stage	Duopolistic	Dasci and Karakul (2009)
Equilibrium model	Simulation	No	General	Identical	Perishable	Dependent	Finite horizon	Oligopolistic	Perakis and Sood (2006)
				Differentiated	Durable	Dependent	Finite horizon	Duopolistic	Adida and Perakis (2010)
				Multi	Durable	Both	1-stage	Duopolistic	Dong et al. (2019)
							1-stage	Oligopolistic	Dong et al. (2019)
							Infinite horizon	Duopolistic	Dong et al. (2019)
							Infinite horizon	Oligopolistic	Dong et al. (2019)
			Transportation	Identical	Perishable	Independent	3-stage	Duopolistic	Netessine and Shumsky (2005)
			Transportation	Differentiated	Perishable	Dependent	Finite horizon	Oligopolistic	Mookherjee and Friesz (2008)
						Independent	3-stage	Duopolistic	Netessine and Shumsky (2005)
						Independent	Infinite horizon	Duopolistic	Isler and Imhof (2008)
	No	Yes	General	Identical	Durable	Independent	1-stage	Oligopolistic	Janssen and Moraga-González (2004)
								Duopolistic	Yano and Komatsubara (2017)
							2-stage	Duopolistic	Yano and Komatsubara (2006)
								Duopolistic	Yano and Komatsubara (2018)
				Differentiated	Durable	Independent	1-stage	Duopolistic	Fay (2008)
									Lal and Sarvary (1999)
				n.a	n.a	n.a	Finite horizon	Duopolistic	Ittoo and Petit (2017)
			Hospitality	Identical	Durable	Independent	4-stage	Oligopolistic	Guo and Zheng (2017)
			Hospitality	Differentiated	Perishable	Independent	2-stage	Duopolistic	Jerath et al. (2010)
			Retail	Identical	Durable	Dependent	Finite horizon	Duopolistic	Babaioff et al. (2015)
						Independent	1-stage	Duopolistic	Balakrishnan et al. (2014)
									Mitra (2021)
									Yao et al. (2005)
Equilibrium model	No	Yes	Retail	Identical	Durable	Indepent	1-stage	Oligopolistic	Balasubramanian (1998)
									Bernstein et al. (2008)
									Koçaş (2005)
							2-stage	Duopolistic	Mitra (2021)
									Viswanathan (2005)
									Yao et al. (2005)
						Independent	3-stage	Duopolistic	Sarkar and Pal (2021)
					n.a	Independent	2-stage	Duopolistic	Wang et al. (2020)
				Differentiated	Durable	Independent	1-stage	Oligopolistic	Oksana (2021)
					Both	Independent	1-stage	Monopolistic	Cattani et al. (2007)
								Duopolistic	Cattani et al. (2007)
			Transportation	Identical	Durable	Dependent	Finite horizon	Duopolistic	Babaioff et al. (2015)
		No	General	Both	Durable	Both	n.a	Oligopolistic	Stiglitz (1979)
						Dependent	3-stage	Oligopolistic	Dana and Williams (2022)
							Infinite horizon	Oligopolistic	Weintraub et al. (2008)
						Independent	1-stage	Duopolistic	Hamilton and Slutsky (1990)
				Identical	Durable	Dependent	2-stage	Oligopolistic	Anton et al. (2014)
							Finite horizon	Duopolistic	Dudey (1992)
							Infinite horizon	Duopolistic	Maskin and Tirole (1988)
						Independent	1-stage	Duopolistic	Matsubayashi and Yamada (2008)
								Oligopolistic	Salop (1976)
							2-stage	Duopolistic	Chen (1997)
									Chioveanu and Zhou (2013)
								Oligopolistic	Boccard and Wauthy (2000)
									Chioveanu and Zhou (2013)
				Differentiated	Durable	Independent	1-stage	Monopolistic	Chen and Riordan (2007)
Equilibrium model	No	No	General	Differentiated	Durable	Independent	1-stage	Oligopolistic	Chen and Riordan (2007)
									Chioveanu (2012)
									Gallego et al. (2006)
							2-stage	Duopolistic	Motta (1993)
					Perishable	Dependent	Infinite horizon	Duopolistic	Villas-Boas (1999)
									Villas-Boas (2006)
						Independent	2-stage	Duopolistic	Lin et al. (2014)
									Villas-Boas (2004)
							3-stage	Duopolistic	Anand and Giotra (2007)
					n.a	Independent	1-stage	Oligopolistic	Aksoy-Pierson et al. (2013)
					n.a	Independent	2-stage	Duopolistic	Moorthy (1988)
			Newsvendor	Identical	Perishable	Independent	1-stage	Duopolistic	Lippman and McCardle (1997)
								Oligopolistic	Lippman and McCardle (1997)
							2-stage	Duopolistic	Lippman and McCardle (1997)
								Oligopolistic	Lippman and McCardle (1997)
			Retail	Identical	Durable	Dependent	3-stage	Duopolistic	Geng and Mallik (2007)
						Independent	1-stage	Duopolistic	Cachon and Harker (2002)
								Oligopolistic	Bernstein and Federgruen (2005)
							2-stage	Duopolistic	Cachon and Harker (2002)
							3-stage	Duopolistic	Wang et al. (2017)
							Infinite horizon	Oligopolistic	Bernstein and Federgruen (2004)
					Perishable	Independent	1-stage	Duopolistic	Parlar (1988)
				Differentiated	Durable	Dependent	3-stage	Duopolistic	Afèche et al. (2011)
Equilibrium model	No	No	Retail	Differentiated	Durable	Independent	1-stage	Duopolistic	Choi et al. (1996)
							2-stage	Duopolistic	Gupta et al. (2020)
									Sun and Gilbert (2019)
							3-stage	Duopolistic	Gupta et al. (2020)
					Perishable	Independent	1-stage	Duopolistic	Wang and Hu (2014)
							2-stage	Duopolistic	Wang and Hu (2014)
				Multi	Durable	Independent	1-stage	Duopolistic	Besbes and Sauré (2016)
									Nip et al. (2021)
								Oligopolistic	Besbes and Sauré (2016)
									Federgruen and Hu (2015)
									Nip et al. (2021)
			Transportation	Differentiated	Perishable	Independent	2-stage	Duopolistic	Zhao et al. (2017)
Pricing model	Yes	Yes	Hospitality	Differentiated	Perishable	Dependent	Infinite horizon	Oligopolistic	Li et al. (2017)
			Retail	Identical	Durable	Dependent	Finite horizon	Oligopolistic	Cao and Gruca (2003)
		No	General	Differentiated	Durable	Dependent	Infinite horizon	Monopolistic	Kachani and Shmatov (2010)
								Oligopolistic	Kachani and Shmatov (2010)
				Multi	Durable	Dependent	Infinite horizon	Monopolistic	Kachani and Shmatov (2010)
			General	Multi	Durable	Dependent	Infinite horizon	Oligopolistic	Kachani and Shmatov (2010)
					n.a	Independent	Finite horizon	Duopolistic	Blattberg and Wisniewski (1989)
			Retail	Identical	Perishable	Dependent	Finite horizon	Oligopolistic	Schlosser and Boissier (2018)
				Differentiated	Durable	n.a	Finite horizon	Duopolistic	Aggarwal and Cha (1998)
				Multi	Durable	Independent	2-stage	Oligopolistic	Dickson and Urbany (1994)
	Simulation	Yes	Retail	Identical	Durable	Both	Infinite horizon	Duopolistic	Wang and Sun (2020)
Pricing model	Simulation	Yes	Retail	Identical	Durable	Dependent	Finite horizon	Duopolistic	Van de Geer et al. (2019)
								Oligopolistic	Van de Geer et al. (2019)
							Infinite horizon	Duopolistic	Schlosser and Richly (2019)
				Differentiated	Perishable	Dependent	Finite horizon	Oligopolistic	Levin et al. (2009)
		No	General	Differentiated	n.a	Dependent	Finite horizon	Oligopolistic	Serth et al. (2017)
				Identical	n.a	Dependent	Finite horizon	Duopolistic	Cooper et al. (2015)
				Differentiated	Durable	Dependent	Infinite horizon	Monopolistic	Tuinstra (2004)
				Differentiated	Durable	Dependent	Infinite horizon	Oligopolistic	Tuinstra (2004)
					n.a	Dependent	Infinite horizon	Oligopolistic	Chung et al. (2012)
			Retail	Identical	Durable	Dependent	Finite horizon	Duopolistic	Anderson et al. (2005)
				Differentiated	Durable	Dependent	Finite horizon	Duopolistic	Liu and Zhang (2013)
								Monopolistic	Liu and Zhang (2013)
			Transportation	Identical	Perishable	Dependent	Finite horizon	Duopolistic	Currie et al. (2008)
	No	Yes	General	Identical	Durable	Dependent	1-stage	Duopolistic	Popescu (2015)
								Oligopolistic	Popescu (2015)
				Identical	Durable	Dependent	2-stage	Duopolistic	Anderson and Kumar (2007)
							Infinite horizon	Duopolistic	Anderson and Kumar (2007)
									Miklós-Thal and Tucker (2019)
									Popescu (2015)
								Oligopolistic	Popescu (2015)
							Finite horizon	Duopolistic	Talluri and Martinez-de-Albeniz (2011)
								Oligopolistic	Talluri and Martinez-de-Albeniz (2011)
								Perfect comp	Yang and Xia (2013)
Pricing model	No	Yes	General	Differentiated	Perishable	Dependent	Finite horizon	Duopolistic	Talluri and Martinez-de-Albeniz (2011)
								Oligopolistic	Talluri and Martinez-de-Albeniz (2011)
			Hospitality	Differentiated	Perishable	Dependent	Finite horizon	Duopolistic	Gallego and Hu (2014)
								Oligopolistic	Gallego and Hu (2014)
			Retail	Identical	Durable	Independent	1-stage	Oligopolistic	Ryan et al. (2012)
				Differentiated	Durable	Independent	1-stage	Duopolistic	Abhishek et al. (2016)
		No	General	Identical	Both	Dependent	Infinite horizon	Duopolistic	Schlosser and Boissier (2017)
					Durable	Dependent	Finite horizon	Duopolistic	Wenzelburger (2004)
								Oligopolistic	Yang et al. (2020)
						Independent	2-stage	Duopolistic	Caillaud and De Nijs (2014)
							Infinite horizon	Duopolistic	Caillaud and De Nijs (2014)
					Perishable	Dependent	Finite horizon	Duopolistic	Mantin et al. (2011)
									Bertsimas and Perakis (2006)
					Perishable	Dependent	Finite horizon	Oligopolistic	Bertsimas and Perakis (2006)
					n.a	Independent	1-stage	Monopolistic	Allen and Hellwig (1986)
					n.a	Independent	1-stage	Oligopolistic	Allen and Hellwig (1986)
								Perfect comp	Allen and Hellwig (1986)
				Differentiated	Durable	Independent	2-stage	Duopolistic	Caplin and Nalebuff (1991)
								Oligopolistic	Caplin and Nalebuff (1991)
					n.a	Dependent	2-stage	Oligopolistic	Gallego and Wang (2014)
Pricing model	No	No	General	n.a	n.a	Dependent	Infinite horizon	Duopolistic	Wu and Wu (2016)
			Retail	Identical	Durable	Independent	1-stage	Duopolistic	Nalca et al. (2010)
Empirical study	Yes	Yes	Automotive	Differentiated	Durable	Dependent	Finite horizon	Oligopolistic	Won et al. (2022)
				Differentiated	Durable	Independent	Finite horizon	Oligopolistic	Netzer et al. (2012)
			Pharmaceutical	Differentiated	Durable	Independent	Finite horizon	Oligopolistic	Netzer et al. (2012)
			Retail	Identical	Durable	Dependent	Finite horizon	Oligopolistic	Wang et al. (2021)
				Differentiated	Durable	Independent	Finite horizon	Oligopolistic	Ringel and Skiera (2016)
				Multi	Both	Dependent	Finite horizon	Oligopolistic	Cavallo (2018)
		No	Airline	Identical	Perishable	Dependent	Finite horizon	Oligopolistic	Siegart and Ulbricht (2020)
				Differentiated	Perishable	Dependent	Finite horizon	Duopolistic	Borenstein and Rose (1994)
								Monopolistic	Borenstein and Rose (1994)
			Automotive	Differentiated	Durable	Dependent	Finite horizon	Oligopolistic	Olivares and Cachon (2009)
						Independent	Finite horizon	Oligopolistic	DeSarbo and Grewal (2007)
			Gasoline	Identical	Durable	Dependent	Finite horizon	Oligopolistic	Noel (2007)
			Retail	Multi	Perishable	Dependent	Finite horizon	Oligopolistic	Putsis and Dhar (1998)

## Footnotes

1 Competitive pricing includes all activities and processes to price products with the consideration of competitors. This does not only include rigidly pegging prices to competitor prices but rather a comprehensive consideration of current and expected price (re-)actions of competing firms to sustainably ensure profit maximization. In this article, the terms competitive pricing, competitor-oriented pricing and competitor-based pricing are used synonymously.

2 Search costs are defined as the costs of time and resources to acquire information with respect to price, assortment, and quality characteristics of the goods provided by different sellers. The internet dramatically reduces search costs through price comparison websites such as Google Shopping, Shopzilla (USA) or Idealo (Germany).

3 Business-to-consumer (B2C) online retail sales encompass all forms of electronic commerce markets in which residential end customers can directly buy goods from a seller over the internet through a web browser or a mobile app. In this paper, the terms business-to-consumer (B2C) online goods retail, online retail, e-tail, e-retail and e-commerce markets are used synonymously.

4 In contrast to classical quantity-based revenue management, dynamic pricing, also known as surge pricing, is the practice of adjusting prices according to current market demand (Boer 2015a). Revenue management, also known as yield management, is a type of price discrimination which originates from the airline and hospitality industries. Typically, revenue management models assume fixed capacities, low marginal cost, varying demand and highly perishable inventory (Talluri and van Ryzin 2004).

5 Keywords used for abstract, title and keyword screening were “competitive pricing”, “competitor-based pricing”, “competition” AND “pricing”. To find literature for online pricing in particular, the search was combined with the keywords “online”, “e-retail”, “ecommerce” and “e-commerce”. Whereas the combination was scanned in great depths, the three competitive keywords were screened for influential papers with implications for online markets.

6 Bertrand competition is a simplified model of competition to explain price competition among (at least) two firms for an identical product at equal unit cost of production. Prices are set simultaneously, and consumers buy without search costs from the firm with the lowest price. When all firms charge the same price, consumer demand is split evenly between firms. A firm is willing to supply unlimited amounts of quantities above the unit cost of production and is indifferent to supply at unit cost as it will earn zero profit. The only Bertrand equilibrium exists when prices are equal to unit cost (i.e., competitive price) as each firm otherwise would have an incentive to undercut all other competitors and thereby rake in the entire market demand. Therefore, there can be no equilibrium at prices above the competitive price and price dispersion cannot occur.

7 In vertical differentiation, consumer choice depends on specific quality levels of product attributes. At the same price, all consumers prefer one product over other products, for example because of superior design. In the simplest form, products differ in one attribute and customers are willing to pay marginal increments of this attribute.

8 In horizontal differentiation, consumer choice depends on preferences for products. At the same price, some customers would buy one product and others other products.

9 We consider product differentiation only to product-related differentiation attributes. However, in competitive pricing literature firm-related differences such as firm loyalty or distribution channels are occasionally attributed to differentiation. For example, Abhishek et al. (2016) differentiate online distribution channels of otherwise homogeneous products and firms.

10 Heterogeneous customer preferences are a key requirement for product differentiation, otherwise price constitutes the only driver of the buying decision (Li et al. 2017). Without heterogeneity in consumers’ marginal willingness to pay for different levels of product quality, there can be no product differentiation (Pigou 1920).

11 Accessed 14–03-2022.

12 Salvage value is defined as the residual cash-flow of a good after its expiration date.

13 For example, game settings on the foundation of Stackelberg games necessarily comprise ≥ 2 stages (Geng and Mallik 2007; Gupta et al.; Wang et al. 2020; Yao and Liu 2005). Another example would be Anand and Girotra (2007) who propose a 3-stage model in which they include the supply chain configuration and determination of production quantities in addition to the actual price setting.

14 As such, we classify n-stage models as dynamic models when not all individual stages serve a specific time-independent purpose.

15 For instance, the Brandenburg consumer advice center (Verbraucherzentrale Brandenburg) examined dynamic price differentiation in online retail and found that 15 of the 16 observed German online shops dynamically changed their prices in 2018 (Dautzenberg et al. 2018).

16 A typical rule would be to set prices always x% lower than competitor prices up to a certain profit threshold.

17 Observable market data include price and stock levels of competitors (Fisher et al. 2018) or clickstream and keyword data of customers (Li et al. 2017).

18 Examples for support programs by online marketplaces are Amazon’s Seller Central (https://sellercentral.amazon.com/gp/help/external/G201994820?language=en_US&ref=efph_G201994820_cont_43381; Accessed 14–03-2022), eBay’s Seller Tools (https://pages.ebay.com/sell/automation.html; Accessed 14–03-2022) or Idealo’s Partner Program (https://partner.idealo.com/de; Accessed 14–03-2022).

19 Cumulatively, these values exceed 100% as some articles discussed more than one kind of market structure. Applied by economists to simplify real markets as the foundation of price theory, perfect competition relates to a market structure which is controlled entirely by market forces and not by individual firms. Instead, individual firms only act as price takers and cannot earn any economic profit. The conditions for a perfect competition, such as full information, homogeneous products, fully rational buyers, no scale, network or externality effects, no entry barriers, and no transaction costs, are rarely attainable in practical settings (Stigler 1957). If not all conditions for perfect competition are fulfilled, the market structure is imperfect which applies to most practical settings. Besides a monopoly with only one seller on the market, three market structures with competing firms exist: Monopolistic, duopolistic, and oligopolistic competition. An oligopoly is characterized by a small number of firms in which the behavior of one firm drives the actions of other firms. A duopoly is a particular case of an oligopoly in which two firms control the market. An extreme case of imperfect competition is (quasi) monopolistic competition in which products are differentiated and firms maintain a certain spare capacity giving them a certain degree of pricing power to maximize their (short-term) profits. In consequence, prices can be higher than corresponding the competitive (Bertrand) price (Vives 2001).

20 For an overview of the top 15 online shops in the United States in 2021, see Davidkhanian (2021).
